# EWSR1-FLI1 expression combined with monoallelic EWSR1 depletion is associated with mitotic spindle damage and nocodazole sensitivity

**DOI:** 10.1242/bio.062763

**Published:** 2026-10-23

**Authors:** Evan T. Schulz, Alejandro Parrales, Tomoo Iwakuma, Mizuki Azuma

**Affiliations:** ^1^ Department of Molecular Biosciences, University of Kansas, Lawrence, KS, USA; ^2^ Department of Pediatrics, Children's Mercy Research Institute, Kansas City, MO, USA

**Keywords:** Tubulin, Mitosis, Sarcoma

## Abstract

Microtubule-destabilizing agents (MDAs) and microtubule-stabilizing agents (MSAs) are commonly used chemotherapeutic agents due to their ability to induce cell death by compromising the dynamics of spindles during mitosis. Ewing sarcoma, the second most common pediatric bone cancer, is known to selectively respond to MDAs as a first-line treatment, but not to MSAs. Ewing sarcoma cells carry an aberrant *EWSR1-FLI1* fusion gene and only one wild-type *EWSR1* allele. To investigate the origin of this MDA sensitivity, we used an isogenic cell line carrying a (*AID-EWSR1/wt;EWSR1-FLI1-mCherry/wt*) genotype that enables conditional induction of EWSR1-FLI1 expression (Tet-On system) and EWSR1 knockdown derived from one *EWSR1* allele (auxin-degron system). A combination of EWSR1-FLI1 expression and EWSR1 knockdown enhances apoptosis upon nocodazole treatment, a type of MDA. Our study revealed that the mitotic spindles of Ewing sarcoma cells (A673, RD-ES and SK-ES1) contain elevated levels of tubulin damage, visualized with GTP-tubulin, compared to mesenchymal stem cells. Consistently, the combination of EWSR1-FLI1 expression and EWSR1 knockdown in the isogenic (*AID-EWSR1/wt;EWSR1-FLI1-mCherry/wt*) cell line also leads to an increased incidence of damage in mitotic spindles. Together, we propose that the sensitivity of Ewing sarcoma cells is potentially derived from the increased levels of damage to mitotic spindles caused by EWSR1-FLI1 expression and EWSR1 knockdown.

## Teaser

**Summary:** The combination of EWSR1-FLI1 expression and monoallelic EWSR1 depletion leads to nocodazole sensitivity by increasing mitotic spindle damage.

## INTRODUCTION

Vinca-alkaloid-derived microtubule-destabilizing agents (MDAs), including vincristine, are a common class of chemotherapy drugs that have been used for cancer treatment since the 1960s (Cutts et al., 1960). The clinical effectiveness of MDAs derives from their tubulin-binding activity that causes mitotic spindle destabilization and dynamism defects, compromising mitotic progression and inducing cell death (Perez, 2009; Jordan, 2002). MDAs (e.g. vincristine) are essential to the chemotherapeutic treatment of Ewing sarcoma, a pediatric bone cancer, validated by decades of demonstrated sensitivity and clinical effectiveness (Perez, 2009; Jordan, 2002). In contrast, microtubule-stabilizing agents (MSAs, e.g. paclitaxel) are less effective for Ewing sarcoma patients as a first-line treatment (Kundranda and Niu, 2015; Jain and Kapoor, 2010). Although both drug classes impair mitosis, it is unclear why Ewing sarcoma is more sensitive to MDAs than MSAs. Therefore, our study aims to fill this knowledge gap by elucidating the etiology of Ewing sarcoma's MDA sensitivity.

All Ewing sarcoma patients express an aberrant fusion gene that contains the N-terminal coding sequence of EWSR1 and the C-terminal coding sequence of one of the *ETS* genes (*FLI1*, *ERG*, *ETV1* or *ETV5*) (Delattre et al., 1992; Jeon et al., 1995; Peter et al., 1997). Among these, *EWSR1-FLI1* is the predominant fusion gene occurring in approximately 85% of Ewing sarcoma patients (Delattre et al., 1992, 1994). Our previous studies demonstrated a unique function of EWSR1-FLI1, in which it induces mitotic dysfunction and aneuploidy in human cells and zebrafish (Azuma et al., 2007; Park et al., 2014, 2021; Kim et al., 2023). We also discovered that EWSR1-FLI1 interacts with EWSR1 and induces mitotic dysfunction (Embree et al., 2009). One possible cause of mitotic dysfunction in Ewing sarcoma cells is damage to mitotic spindles, an important type of microtubule defect. This damage results from the emergence of gaps within the spindle lattice due to the detachment of α-tubulin and β-tubulin dimers. Microtubule damage may enhance the sensitivity of spindle fibers to MDAs by compromising fiber stability, dynamism, increasing availability of binding sites for various MDAs or inducing lattice deacetylation (Schaedel et al., 2015; Rai et al., 2021; Igaev and Grubmuller, 2020; Muhlethaler et al., 2021; Andreu-Carbo et al., 2024; Xu et al., 2017). Damage is repaired and stabilized by the incorporation of GTP-tubulin, resulting in islands of GTP-tubulin within the microtubule lattice (Dimitrov et al., 2008). While complete repair of spindle damage can ameliorate tubulin lattice stability, GTP-tubulin is often rapidly hydrolyzed to GDP-tubulin to maintain proper lattice conformation, fiber dynamism, motor protein translocation, and microtubule-associated protein (MAP) interactions (Dimitrov et al., 2008; Aumeier et al., 2016; Triclin et al., 2021; Manka and Moores, 2018; Zehr et al., 2026; Janke and Magiera, 2020; Gudimchuk and McIntosh, 2021). These studies suggest that high levels of GTP-tubulin incorporation in microtubules indicate areas of compromised dynamism in mitotic spindles. While EWSR1 interacts with α-tubulin and is reported to stabilize the non-mitotic tubulin structure, it is unknown how EWSR1 and EWSR1-FLI1 regulate the mitotic spindle structure (Wang et al., 2016). Here, we propose that the sensitization of cells expressing EWSR1-FLI1 along with EWSR1 knockdown results from an increase in tubulin damage that enhances the sensitivity to nocodazole, a type of MDA.

## RESULTS

While sensitivity to MDAs has been established for Ewing sarcoma cells, understanding the cause of this tumor-type-specific sensitivity has been limited. To confirm the cytotoxic effect of MDAs on Ewing sarcoma cells, we employed nocodazole, which depolymerizes spindles by interacting with monomer β-tubulin (Perez, 2009; Jordan, 2002). Ewing sarcoma cells (A673, RD-ES and SK-ES1) and mesenchymal stem cells (MSCs) were treated with nocodazole for 24 h, and the incidence of apoptosis was measured by Annexin V staining. The apoptosis rate of the nocodazole-treated Ewing sarcoma cells (A673, RD-ES and SK-ES1) was significantly higher than that of MSCs, where no significant increase in apoptosis was observed, suggesting that the Ewing sarcoma cells are more sensitive to nocodazole (Fig. 1A) (Jain and Kapoor, 2010). Importantly, nocodazole treatment in MSCs did not induce a significant increase in apoptosis, highlighting the difference in nocodazole sensitivity between Ewing sarcoma and MSCs (Fig. 1A).

**Fig. 1.**
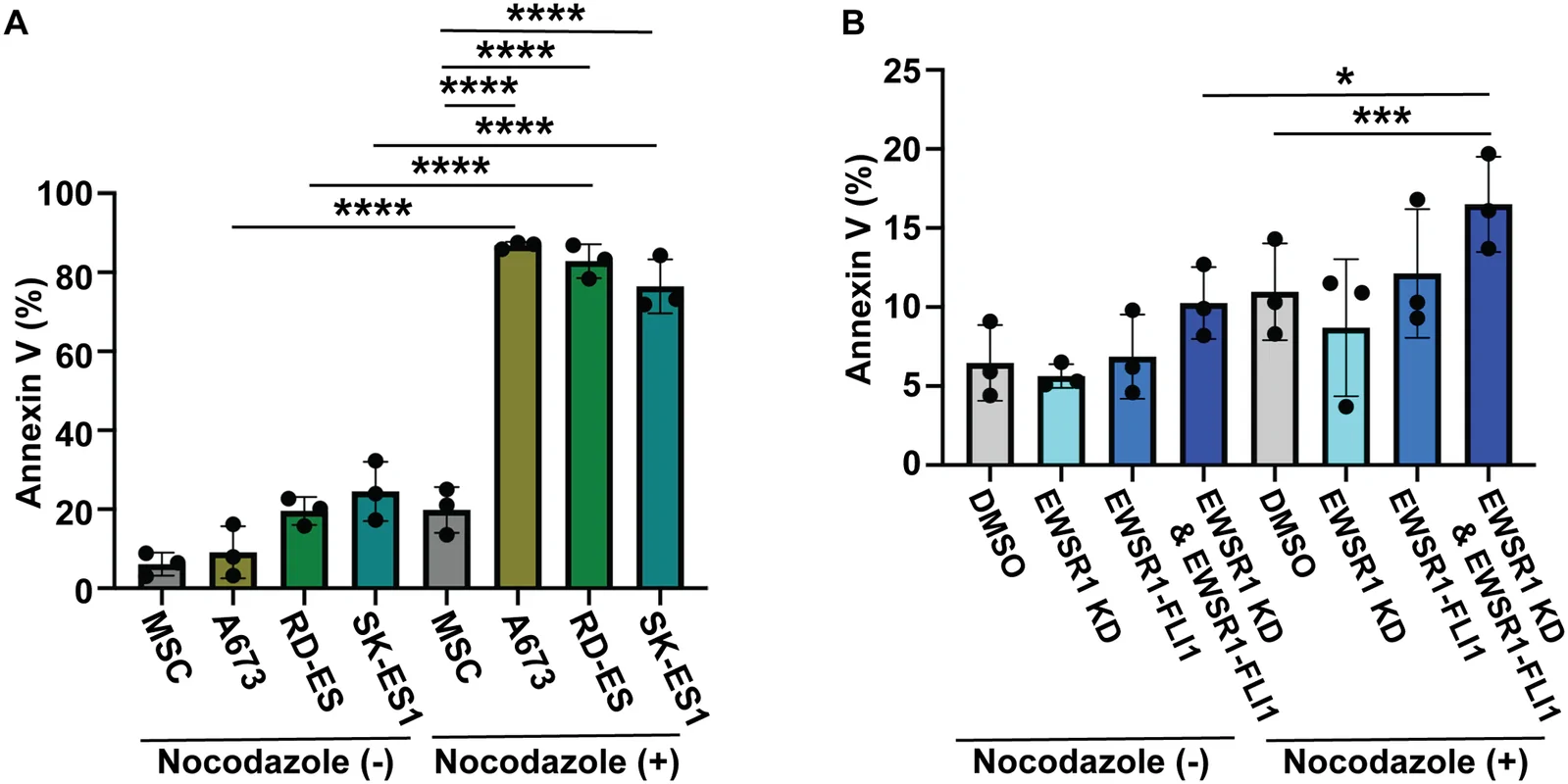
**Nocodazole treatment induces an increased incidence of apoptosis in Ewing sarcoma cells and DLD-1 cells with EWSR1 knockdown and EWSR1-FLI1 expression.** (A) Percentages of cells undergoing apoptosis were visualized by Annexin V staining in Ewing sarcoma cells (A673, RD-ES, and SK-ES1) and MSCs. (B) Percentages of apoptotic cells were visualized by Annexin V staining in four samples induced in (*AID-EWSR1/wt;EWSR1-FLI1-mCherry/wt*) DLD-1 cells: control (DMSO), EWSR1 knockdown (AUX), EWSR1-FLI1 (DOX) and EWSR1 knockdown/EWSR1-FLI1 (AUX/DOX). A minimum of 50 cells were scored for each experiment, and the experiments were repeated three times for all experiments [degrees of freedom (DF)=7] (A,B). Nocodazole (−): Cells were treated without nocodazole. Nocodazole (+): Cells were treated with 332 nM nocodazole for 24 h. *P<0.05, ***P<0.001, ****P<0.0001. All combinations of the samples not indicated with asterisks in the figure were nonsignificant (NS).

To assess the effect of *EWSR1-FLI1* and the loss of one *EWSR1* allele, we utilized an (*AID-EWSR1/wt;EWSR1-FLI1-mCherry/wt*) cell line that was originally generated using a CRISPR/Cas9 system (Hapugaswatta et al., 2026 preprint). This cell line was genetically engineered with the integration of a Tet-On *EWSR1-FLI1* construct at the safe harbor *AAVS1* locus and the mini-AID sequence at the 5′ end of one *EWSR1* allele (Hapugaswatta et al., 2026 preprint). This cell line enables the conditional expression of EWSR1-FLI1 using the Tet-On system and the conditional degradation of EWSR1 derived from one *EWSR1* allele using the auxin (AUX)-degron system. The DLD-1 cell line was chosen for the cell line establishment, as it does not express EWSR1-FLI1 and has a near-diploid karyotype (Vigano et al., 2018; Lengauer et al., 1997). The cells were treated with AUX and/or doxycycline (DOX) to degrade EWSR1 derived from one *EWSR1* allele and/or induce EWSR1-FLI1 expression, respectively, and were further treated with the vehicle or nocodazole for 24 h (Fig. S1). These eight samples were subjected to Annexin V staining, and their rates of apoptosis were calculated. Only the group that had EWSR1-FLI1 induction and EWSR1 knockdown displayed significantly higher apoptosis compared with the control group (Fig. 1B). These results suggest that the combination of EWSR1-FLI1 expression and EWSR1 knockdown is required for the acquisition of the nocodazole sensitivity.

Since EWSR1-FLI1 and EWSR1 share the same N-terminus of EWSR1, there is a technical challenge in distinguishing between the two variants using immunocytochemistry in Ewing sarcoma cells. To investigate whether EWSR1-FLI1 and EWSR1 localize at the mitotic spindle, we utilized the (*AID-EWSR1/wt;EWSR1-FLI1-mCherry/wt*) cells (Hapugaswatta et al., 2026 preprint). This cell line was genetically engineered by integrating the mAID-3xFLAG at the 5′ end of one *EWSR1* locus and the *EWSR1-FLI1-mCherry* at the *AAVS1* locus. The cells were subjected to immunocytochemistry using an anti-FLAG antibody to visualize mAID-EWSR1 (green), anti-mCherry to visualize EWSR1-FLI1-mCherry (red) and anti-α-tubulin antibody to visualize mitotic spindle (blue). These cells were imaged using a confocal microscope. Single Z-section images of the cells were analyzed with the image software Fiji to study the colocalization of combinations of EWSR1 and α-tubulin (Pearson's coefficient: 0.76±0.04), EWSR1-FLI1 and α-tubulin (Pearson's coefficient: 0.80±0.04), and EWSR1-FLI1 and EWSR1 (Pearson's coefficient: 0.77±0.06) (Fig. S2A-C). While EWSR1 colocalizes with EWSR1-FLI1-mCherry throughout the cell as expected, we discovered that both molecules also localize on mitotic spindles in the (*AID-EWSR1/wt;EWSR1-FLI1-mCherry/wt*) cells.

Previous studies have shown that EWSR1 interacts with α-tubulin and stabilizes microtubules during the non-mitotic phase, whereas EWSR1 knockdown destabilizes the spindle structure (Wang et al., 2016). Given the known dominant-negative effect of EWSR1-FLI1 on EWSR1, the combination of EWSR1 knockdown and EWSR1-FLI1 expression would be expected to induce damage to mitotic spindles. While high levels of GTP-tubulin are known to localize to the GTP cap of mitotic spindles and stabilize the spindles during normal mitosis, an increase in GTP-tubulin antibody signal has been used as a well-established marker of mitotic spindle damage. Therefore, we hypothesized that the combination of EWSR1-FLI1 expression and EWSR1 knockdown would induce damage in mitotic spindles. To test this, we conducted immunocytochemistry using anti-GTP-tubulin (green) and anti-α-tubulin (blue) antibodies in (*AID-EWSR1/wt;EWSR1-FLI1-mCherry/wt*) cells treated with AUX and/or DOX for 24 h and 7 days (Fig. 2A,B). The intensity of the signals for anti-α-tubulin and GTP-tubulin was measured (a minimum of 30 cells per sample, and the experiment was repeated for *n*=3 times), and the signals of GTP-tubulin were normalized against the signals of α-tubulin (Fig. 2C,D). GTP-tubulin signals were significantly higher in both cells with EWSR1 knockdown (AUX) and cells with EWSR1-FLI1 expression (DOX) than in control cells (DMSO) after 24 h and 7 days (Fig. 2C,D). Moreover, the combination of EWSR1 knockdown and EWSR1-FLI1 expression (AUX/DOX) displayed a higher GTP-tubulin signal than either EWSR1 knockdown (AUX) or EWSR1-FLI1 expression (DOX) alone, when compared to the control (DMSO) after 24 h and 7 days (Fig. 2C,D). These results support a role for EWSR1 in safeguarding mitotic spindle integrity and reveal a cooperative interaction between EWSR1 depletion and EWSR1-FLI1 expression in inducing mitotic spindle damage. Although the (*AID-EWSR1/wt;EWSR1-FLI1-mCherry/wt*) DLD-1 cell does not share the same cell of origin as Ewing sarcoma, this isogenic cell line exhibited increased mitotic spindle damage when the Ewing sarcoma-associated aberrations were induced. Since the process of mitosis is largely conserved across cell types, this isogenic cell line may serve as an initial model for investigating the monoallelic activity of EWSR1 and/or EWSR1-FLI1 expression during mitosis.

**Fig. 2.**
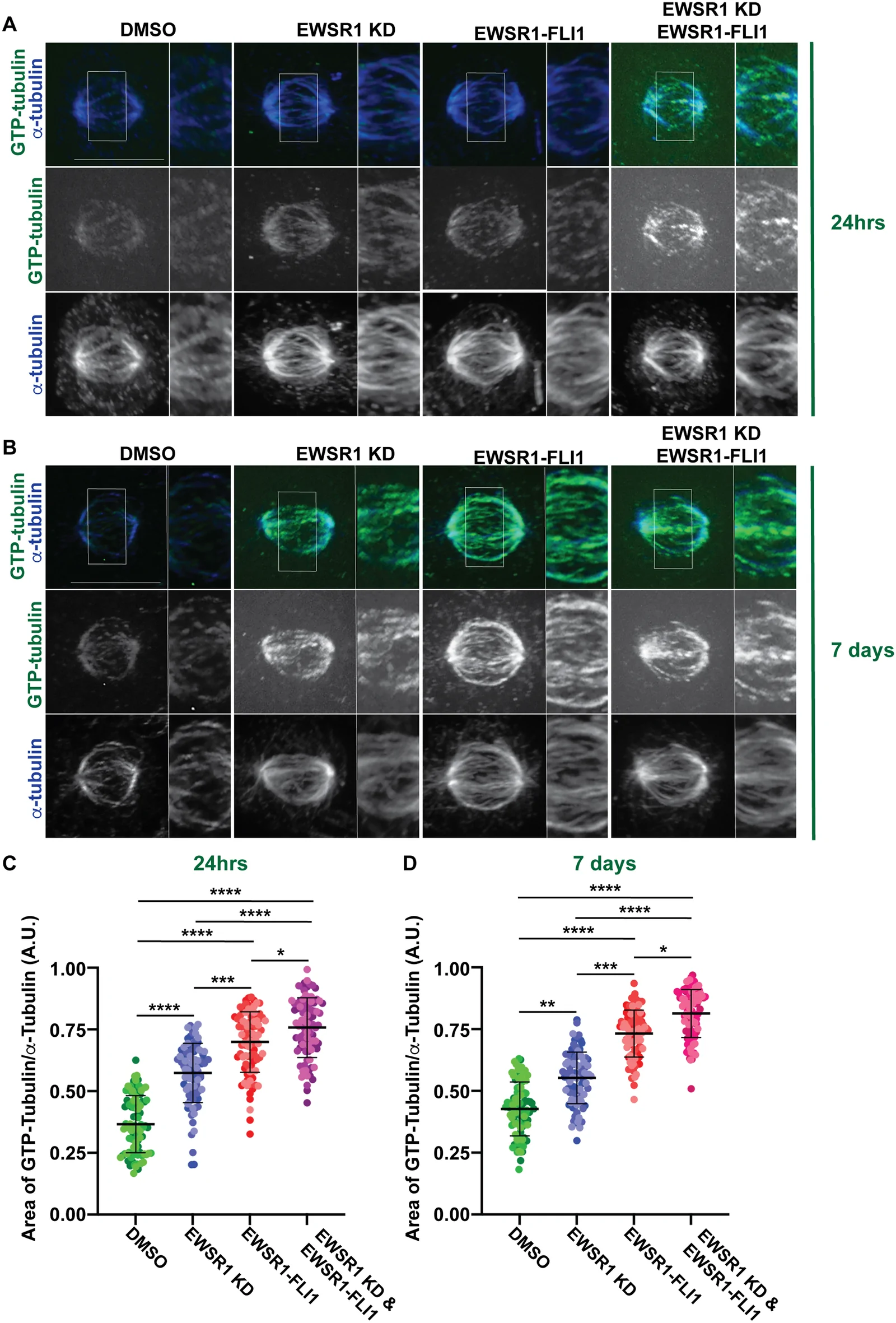
**A combination of EWSR1 knockdown and EWSR1-FLI1 expression results in the induction of damage to microtubules.** (A,B) Representative images of metaphase spindles of (*AID-EWSR1/wt;EWSR1-FLI1-mCherry/wt*) DLD-1 cells. The cells were visualized with anti-α-tubulin (blue) and anti-GTP-tubulin (green) obtained from control (DMSO), EWSR1 knockdown (AUX), EWSR1-FLI1 expression (DOX), and EWSR1 knockdown/EWSR1-FLI1 expression (AUX/DOX). Scale bar: 10 μm. (C,D) The area of GTP-tubulin signals was normalized against the area of α-tubulin. A minimum of 30 cells per sample were quantified, and the experiment was repeated for *n*=3 experiments (DF=3), (A,C) were treated with AUX and/or DOX for 24 h, and (B,D) were treated with AUX and/or DOX for 7 days. **P<0.01, ****P<0.0001, NS: nonsignificant.

To investigate whether Ewing sarcoma cells exhibit high levels of GTP-tubulin in mitotic spindles, we performed immunocytochemistry using anti-GTP-tubulin (green) and anti-α-tubulin (blue) antibodies in Ewing sarcoma cells (A673, RD-ES and SK-ES1). We then compared its intensity of staining with that of MSCs, which are among the proposed cells of origin of Ewing sarcoma (Fig. 3A) (Tirode et al., 2007). The intensity of GTP-tubulin signals in Ewing sarcoma cells was significantly higher than that of MSCs (a minimum of 20 cells per sample were quantified across *n*=3 experiments) (Fig. 3B). It should be noted that the intensity of GTP-tubulin signals was significantly higher in A673 cells than in RD-ES and SK-ES1 cells, though the mechanism is unknown, and the variation may represent differences in the degree of damage among the cell lines. Together, these results indicate that the GTP-tubulin levels are commonly higher in Ewing sarcoma cells than in MSCs, and this difference may explain the increased sensitivity of Ewing sarcoma cells to nocodazole.

**Fig. 3.**
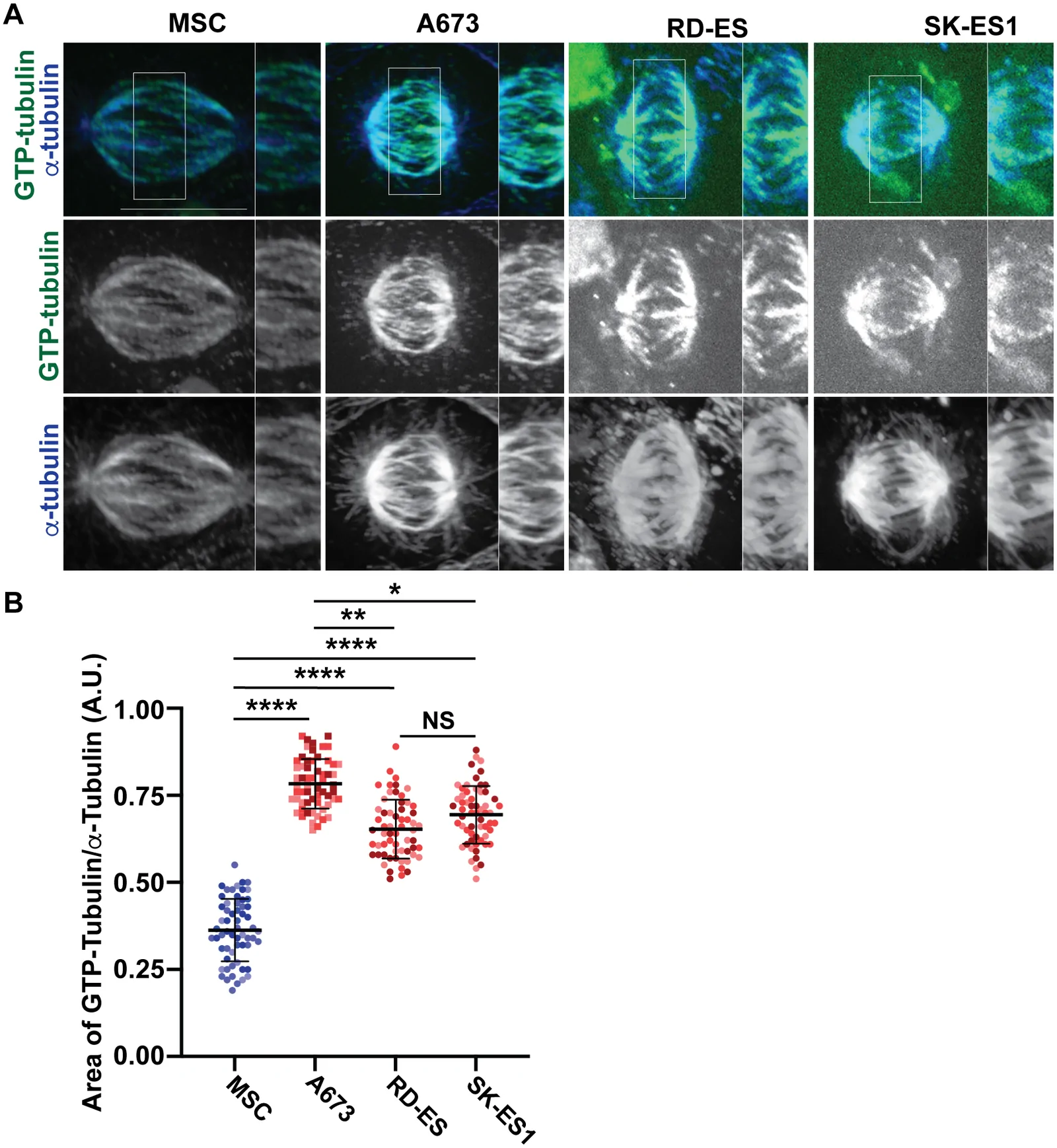
**Ewing sarcoma cells have higher levels of damage to microtubules than do MSCs.** (A) Representative images of metaphase spindles visualized with anti-α-tubulin (blue) and anti-GTP-tubulin (green) in MSCs and A673, RD-ES, and SK-ES1 cells. (B) The intensity of the signals from a minimum of 20 cells was quantified from *n*=3 experiments (DF=3). ****P<0.0001). All combinations of the samples not indicated with asterisks in the figure were nonsignificant (NS). Scale bar: 10 μm.

## DISCUSSION

This study aimed to investigate the tumor-type-specific cause of MDA sensitivity observed in Ewing sarcoma. We discovered that Ewing sarcoma cells exhibit elevated levels of GTP-tubulin in their mitotic spindles compared to MSCs, suggesting an increased level of damage to the mitotic spindles. Ewing sarcoma cells also undergo a higher rate of apoptosis under treatment with nocodazole, a type of MDA, than do MSCs. Furthermore, mimicking the genotype of Ewing sarcoma by knocking down EWSR1 and expressing EWSR1-FLI1 conditionally in DLD-1 cells increased levels of GTP-tubulin in mitotic spindles and the rate of apoptosis under nocodazole treatment. These data suggest that the combination of EWSR1 knockdown and EWSR1-FLI1 expression is sufficient to induce mitotic spindle damage, thereby sensitizing cells to nocodazole-mediated microtubule disruption. However, it remains unclear whether this increased sensitivity to MDA arises primarily from reduced EWSR1 activity resulting from the dominant-negative effect of EWSR1-FLI1 or whether additional functions of EWSR1-FLI1 contribute to the phenotype in cooperation with compromised EWSR1 activity. Nonetheless, our findings support a model in which mitotic spindle damage caused by Ewing sarcoma-specific molecular abnormalities contributes to increased MDA sensitivity.

In this study, the DLD-1 cell line was chosen to conditionally model the development of Ewing-sarcoma-associated aberrations for the following reasons. First, DLD-1 cells are the most frequently used cell line to study the mechanism of aneuploidy induction due to the cell line's near-diploid karyotype (Hapugaswatta et al., 2026 preprint). Therefore, this cell line, which enables conditional induction of EWSR1-FLI1 expression and monoallelic EWSR1 knockdown, enabled us to dissect the mechanisms underlying the induction of mitotic dysfunction and aneuploidy. Second, to understand the direct defects caused by the EWSR1-FLI1 expression and EWSR1 knockdown, conditional induction of EWSR1-FLI1 expression and monoallelic depletion of EWSR1 in a cell line with natively absent EWSR1-FLI1 expression and biallelic EWSR1 were essential to systematically characterize their effects. While the phenotypes between the (*AID-EWSR1/wt;EWSR1-FLI1-mCherry/wt*) DLD-1 cell line and Ewing sarcoma cell lines resemble each other, a cautious verification will be valuable in future studies. The limitation of using the (*AID-EWSR1/wt;EWSR1-FLI1-mCherry/wt*) DLD-1 cell line is derived from differences in cell type and genetic background. To address this limitation, it may be ideal to generate the (*AID-EWSR1/wt;EWSR1-FLI1-mCherry/wt*) system in cells with a normal genetic background and with the same lineage, including MSCs, neural crest cells, or induced pluripotent stem cells in future studies (Tirode et al., 2007; Vasileva et al., 2025).

Damage to the tubulin lattice induces mechanical stress and conformational deformations across the microtubule lattice, causing loss of fiber stability, stiffness, and dynamism, as well as altering MAP interactions and motor protein trafficking (Schaedel et al., 2015; Igaev and Grubmuller, 2020; Triclin et al., 2021; Nandakumar et al., 2026; Kalutskii et al., 2025; Brouhard and Rice, 2018). To further understand how the stability of mitotic spindles is compromised in Ewing sarcoma, mitotic spindle formation rate, dynamism, and posttranslational modification (PTM) need to be characterized by the following means in Ewing sarcoma cells. The damaged tubulin lattice is unstable and remains unstable until the gaps in the lattice are replaced with GTP-tubulin. Therefore, it is plausible that the majority of mitotic spindles in Ewing sarcoma cells are destabilized. If the rate of microtubule formation or dynamic instability is reduced in Ewing sarcoma, it will provide evidence of spindle destabilization and support our observation that Ewing sarcoma is sensitized to MDAs. Additionally, the PTM status of mitotic spindles can enhance their stability. A previous study demonstrated that the EWSR1 promotes acetylation of spindles through the modulation of histone deacetylase 6 (Wang et al., 2016). Therefore, spindle acetylation should be quantified in Ewing sarcoma cells, as it is plausible that the loss of the *EWSR1* allele affects the rate of spindle acetylation and destabilizes mitotic spindles.

In this study, we observed that the levels of GTP-tubulin in A673 cells were higher than in RD-ES and SK-ES1 cells, suggesting a variation in the degree of intrinsic tubulin damage among Ewing sarcoma tumors. One possible explanation for the differences is the presence of polymorphisms in *α-tubulin* and *β-tubulin* genes. For example, the T238A mutation in human β-tubulin stabilizes tubulin by altering the rate of GTP uncoupling through its conformational change within the microtubule (Li et al., 2020). The H283Y mutation in α-tubulin was proposed to stabilize tubulin filaments by promoting the interaction between interdimer, intradimer and lateral interactions between protofilaments (Hari et al., 2003). Therefore, polymorphisms in *tubulin* genes could affect MDA sensitivity or resistance by directly modifying spindle stability. Likewise, it will be important to subclassify the types of polymorphisms that are not sensitive to MDAs. Identification and classification of *tubulin* gene polymorphisms in patients could predict the drug efficacy, indicate resistance formation, and inform treatment strategies.

To better understand the MDA sensitivity in Ewing sarcoma, additional factors should be comprehensively investigated in future studies. First, it is important to determine whether other intrinsic defects impact sensitivity to MDAs in Ewing sarcoma. For example, replication stress is a prominent feature in Ewing sarcoma, and it is associated with impaired BRCA1-dependent DNA repair due to increased R-loop formation or stalled replication forks due to STAG2 mutations (Gorthi et al., 2018; Mondal et al., 2019). One study showed that replication stress can be enhanced by inhibition of the ATR-CHK1-WEE1 pathway, leading to the induction of apoptosis without cells entering mitosis (Koppenhafer et al., 2026). Interestingly, mild replication stress can lead to microtubule dysregulation and aneuploidy in other types of cancer. Thus, it is important to assess how different degrees of DNA replication stress, or whether different types of replication stress inducers, affect MDA sensitivity in Ewing sarcoma.

Second, identifying the cell death pathways induced by MDA in treated Ewing sarcoma cells will also be crucial. One attractive candidate mediating MDA sensitivity is FAS/CD95 (Mitsiades et al., 1998). The *FAS/CD95* mRNA undergoes EWSR1-dependent alternative splicing to generate a proapoptotic isoform, but this process is compromised in Ewing sarcoma cells due to *EWSR1* haploinsufficiency (Paronetto et al., 2014). The cyclic GMP-AMP synthase stimulator of interferon genes (STING) pathway is an alternative candidate pathway that may mediate MDA sensitivity because it mediates cell death during mitosis.

Lastly, it is important to consider the tumor microenvironment as a factor that may impair MDA sensitivity in Ewing sarcoma. Differences in MDA sensitivity between monolayer-cultured tumor cells, 3D culture tumoroids, and tumors *in vivo* have been documented, and the biophysical, biochemical, and metabolic complexity of the tumor microenvironment presents many possible pathways that could dynamically alter drug penetration or efflux and tumor gene expression to change MDA sensitivity in patients, with some reported in the context of Ewing sarcoma. Notably, cancer-associated fibroblasts secrete interleukin-6 (IL-6) and reduce MDA sensitivity of Ewing sarcoma cells (Lu et al., 2025). In addition, Ewing sarcoma promotes osteoclast activation by secreting receptor activator of nuclear factor kappa beta (RANKL), IL-6, and tumor necrosis factor alpha, leading to bone loss, which may further impair MDA sensitivity (Redini and Heymann, 2015; Lau et al., 2007). In summary, investigating the aforementioned pathways within the same experimental assay will be essential to gain a comprehensive understanding of the mechanisms underlying MDA sensitivity in Ewing sarcoma.

## MATERIALS AND METHODS

### Cell culture, induction of EWSR1 knockdown and EWSR1-FLI1 expression, and treatment with nocodazole

The Ewing sarcoma cells (A673, RD-ES and SK-ES1) and MSCs were cultured according to the protocol provided by the American Type Culture Collection. As previously described, the (*AID-EWSR1/wt;EWSR1-FLI1-mCherry/wt*) cell line was generated by inserting an *mAID-3xFLAG* DNA construct into the 5′ end of one *EWSR1* locus and an *EWSR1-FLI1-mCherry* DNA construct into the *AAVS1* locus (Hapugaswatta et al., 2026 preprint). The (*AID-EWSR1/wt;EWSR1-FLI1-mCherry/wt*) DLD-1 cell line (OSTIR1-7-21-38) was maintained in McCoy's medium supplemented with 10% Tet-free FBS, 1 μg/ml blasticidin (InvivoGen, #ant-bl), 200 μg/ml Hygromycin B Gold (InvivoGen, #ant-hg), and 1 μg/ml puromycin (Park et al., 2021; Kim et al., 2023; Hapugaswatta et al., 2026 preprint).

To induce EWSR1 knockdown resulting from one *EWSR1* allele and/or EWSR1-FLI1 expression, the cells were subjected to one of the four conditions for 24 h: control (treated with DMSO), EWSR1 knockdown (treated with 1 mM AUX), EWSR1-FLI1 expression (treated with 1 μg/ml of DOX), or EWSR1 knockdown/EWSR1-FLI1 expression (treated with 1 mM AUX and 1 μg/ml of DOX). The (*AID-EWSR1/wt;EWSR1-FLI1-mCherry/wt*) cell line, MSCs and Ewing sarcoma cells were treated with 0.33 μM nocodazole for 24 h. When the (*AID-EWSR1/wt;EWSR1-FLI1-mCherry/wt*) cells were treated with AUX and/or DOX, nocodazole was added concurrently to the culture medium.

### Apoptosis assay

The apoptotic cells were visualized using Annexin V Alexa Fluor 568 according to the protocol provided by Invitrogen (cat. no. A13202). The cells were photographed with EVOS imaging systems (Thermo Fisher Scientific), and the percentage of apoptotic cells was calculated.

### Immunocytochemistry and GTP-tubulin assay

The cells were permeabilized and stained with hMB11 (Adipogen, cat. no. AG-27B-0009-C100, 1:5000 dilution) and antihuman IgG secondary antibodies (Alexa Fluor, cat. no. A11013, 1:1000 dilution), according to a previously established protocol with the following minor modifications (de Forges et al., 2013). For all α-tubulin co-labeling steps, PEM-G (80 mM PIPES, 0.5 mM EDTA, 2 mM MgCl_2_, 10% glycerol) was used instead of PBS. To visualize mitotic spindles, EWSR1 and EWSR1-FLI1-mCherry, the cells were treated with rabbit anti-α-tubulin (Sigma-Aldrich, cat. no. 11224-1-AP, 1:500 dilution), mouse anti-EWSR1 (Abnova, cat. no. H00002130-M01, 1:500 dilution) and chicken anti-mCherry (LSBio, cat. no. LS-C204825, 1:500 dilution) for 3 h at room temperature. The cells were subjected to the following secondary antibodies at room temperature for 3 h: goat anti-rabbit IgG Alexa Fluor 405 (Invitrogen, cat. no. A-31556, 1:250 dilution), goat anti-mouse IgG Alexa Fluor 488 (Invitrogen, cat. no. A-32723, 1:250 dilution), and goat anti-chicken IgG Alexa Fluor 568 (Invitrogen, cat. no. A-11041, 1:250 dilution). The cells were washed three times with 4°C PEM-G for 5 min at room temperature and mounted onto slides with antifade mounting medium (VECTASHIELD, cat. no. H-1000).

### Documentation of cells and image analysis

A minimum of 20 metaphase cells were imaged from each experiment (*N*=3) by acquiring Z-section images at 0.2 μm intervals, using a spinning disk confocal microscope (Nikon, Crest Optics X-Light V3 spinning disk confocal system on an Eclipse Ti2-E). Image analysis was conducted using the Fiji image software by selecting the region of interest (ROI) corresponding to the mitotic spindle using the freehand selection tool based on the α-tubulin staining. The channels containing α-tubulin and GTP-tubulin signals were separated, and the percentage of overlap between the two was calculated using the JACoP plug-in. This confocal imaging was performed in the Microscopy and Analytical Imaging Core Laboratory at the University of Kansas.

To quantify the degree of FLAG-EWSR1 knockdown and EWSR1-FLI1-mCherry expression, a minimum of 100 cells were imaged from each experiment (*N*=3 in each treatment group) using a fluorescence microscope (Nikon, Eclipse Ti). Image analysis was conducted using the Fiji image software. First, the image channels containing the DAPI, FLAG-EWSR1, and EWSR1-FLI1-mCherry signals were separated. Then the background of the EWSR1 (tagged with FLAG) and EWSR1-FLI1 (fused to mCherry) channels was subtracted using the Subtract Background function in Fiji, with a rolling-ball radius of 50 pixels. The split channel images were then merged, and the ROI corresponding to nuclear DNA was selected using the freehand selection tool based on DAPI staining. The mean EWSR1 and EWSR1-FLI1 signal intensity of the defined ROI was then measured using the Fiji measure function.

### Statistics

The error bars depicted in each graph represent the s.d. Statistical significance was defined as *P*<0.05 using a two-way analysis of variance, which compared the nested replicate means across each group (*N*=3), with subsequent post hoc analysis conducted using Tukey's honestly significant difference test.

## Supplementary Material



10.1242/biolopen.062763_sup1 Supplementary information
