# Supplementary material for: EWSR1-FLI1 expression combined with monoallelic EWSR1 depletion is associated with mitotic spindle damage and nocodazole sensitivity
Source: Biol Open. 2026 Oct 23;15(10):bio062763. doi: 10.1242/bio.062763 (PMC13730126; doi:10.1242/bio.062763)
Supplement: Supplementary file 1 [file biolopen-15-062763-s1.pdf]

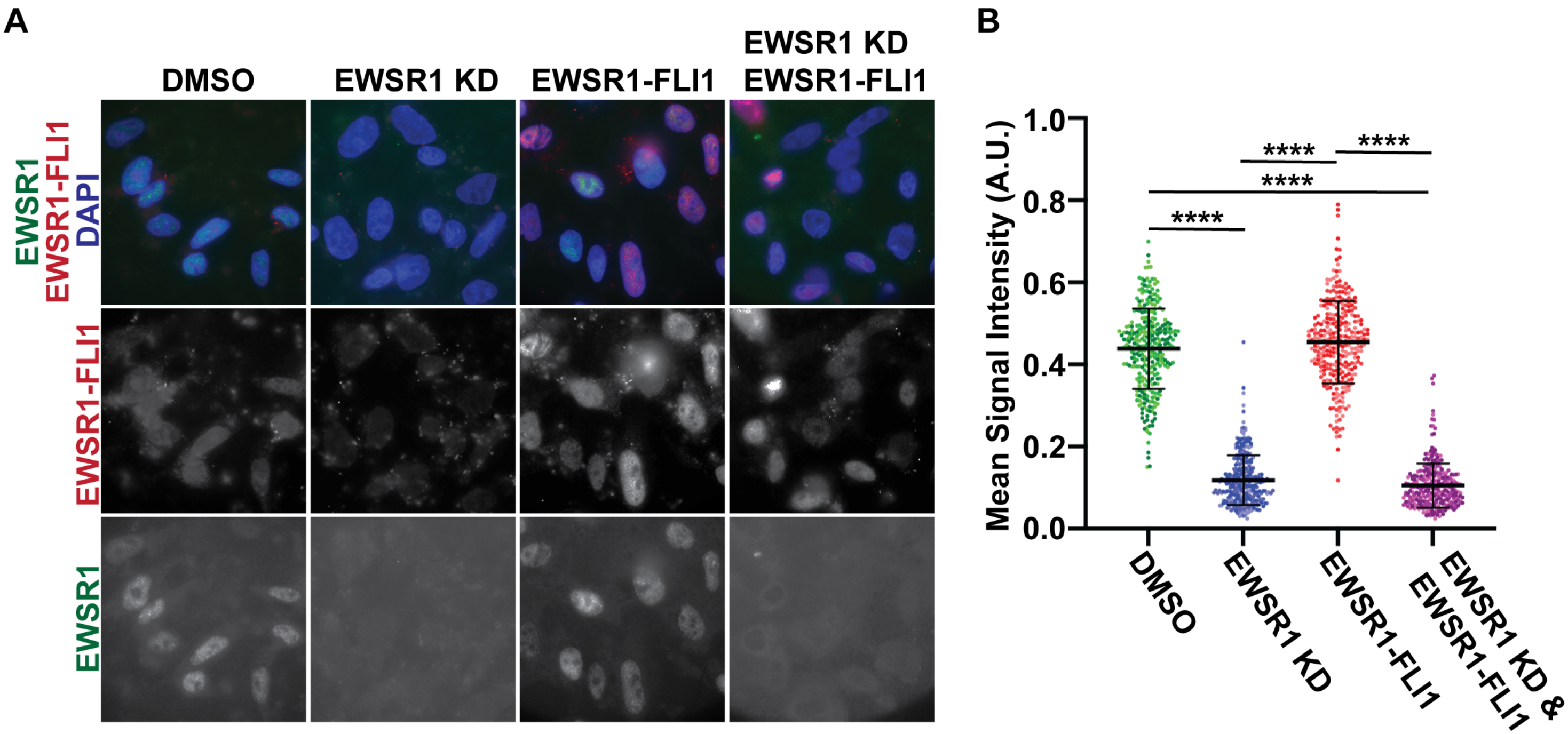


**Fig. S1.**

Biology Open • Supplementary information

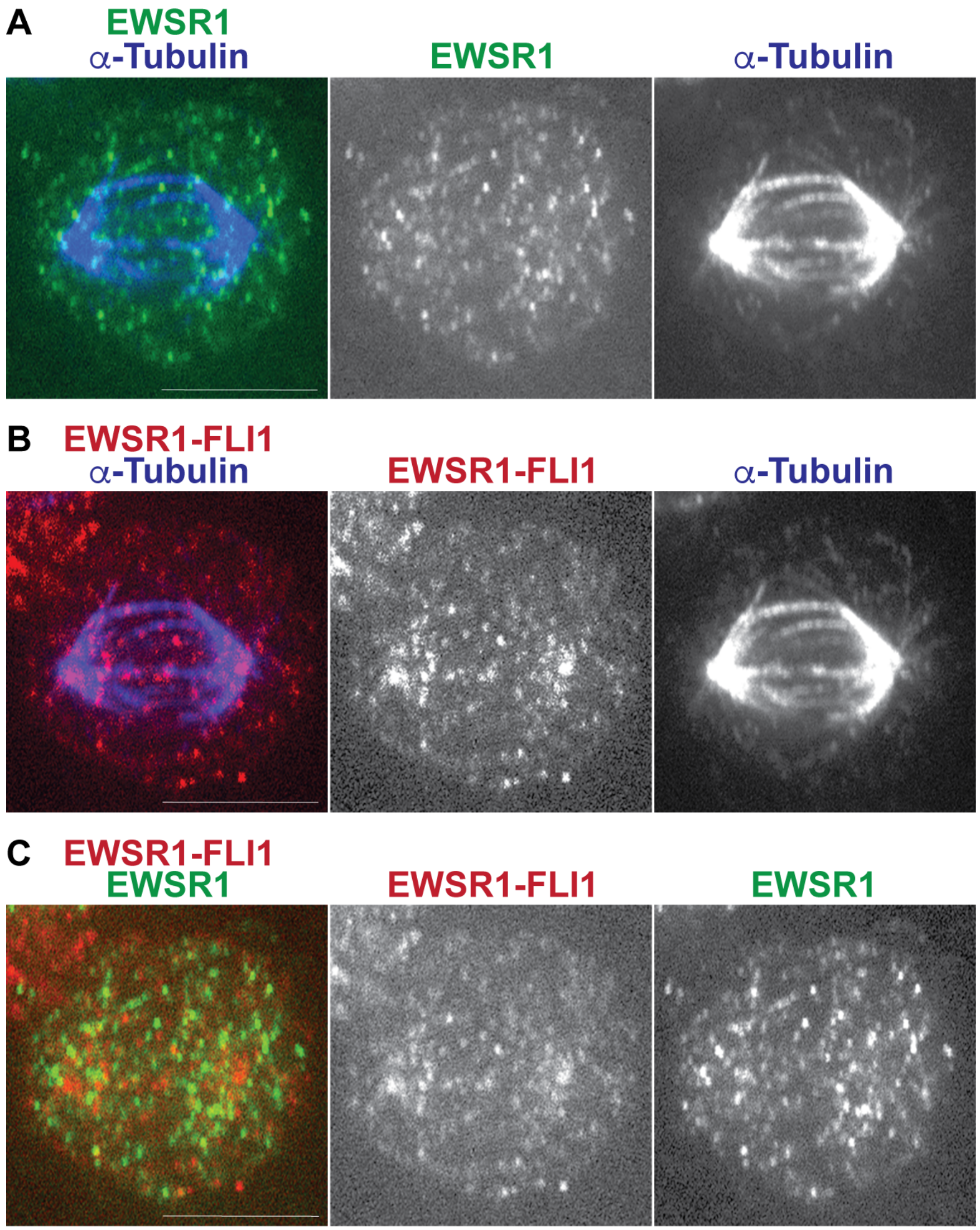
A
EWSR1
α-Tubulin
EWSR1
α-Tubulin
B
EWSR1-FLI1
α-Tubulin
EWSR1-FLI1
α-Tubulin
C
EWSR1-FLI1
EWSR1
EWSR1-FLI1
EWSR1

**Fig. S2.**
