# Supplementary material for: EWSR1-FLI1 expression combined with monoallelic EWSR1 depletion is associated with mitotic spindle damage and nocodazole sensitivity
Source: Biol Open. 2026 Oct 23;15(10):bio062763. doi: 10.1242/bio.062763 (PMC13730126; doi:10.1242/bio.062763)
Supplement: Supplementary file 2 [file biolopen-15-062763_review_history.pdf]

EWSR1-FLI1 expression combined with monoallelic EWSR1 depletion is associated with mitotic spindle damage and nocodazole sensitivity

Evan T. Schulz, Alejandro Parrales, Tomoo Iwakuma and Mizuki Azuma

10.1242/bio.062763

Editor: Christopher Maher

Review timeline

Original submission:	22 June 2026
Editorial decision:	30 June 2026
First revision received:	12 August 2026
Editorial decision:	20 August 2026
Second revision received:	6 September 2026
Accepted:	10 September 2026

Original submission

First decision letter

MS ID#: bio.062763

MS Title: EWSR1-FLI1 expression combined with monoallelic EWSR1 depletion is associated with mitotic spindle damage and nocodazole sensitivity

Authors: Evan T. Schulz; Mizuki Azuma

Dear Dr Azuma,

I have now reached a decision on the above manuscript.

The reviewer reports are shown at the bottom of this email.

As you will see, the reviewers raised a number of substantial criticisms that prevent me from accepting the paper at this stage.

They suggest, however, that a revised version might prove acceptable, if you can address their concerns. If you think that you can deal satisfactorily with the criticisms on revision, I would be pleased to see a revised manuscript. We would then return it to the reviewers.

At this stage, we also ask you to ensure your manuscript complies with our formatting guidelines. Provided you are able to fully address the referees' comments, we are positive about publication of your paper (we accept over 95% of revision submissions) and therefore hope you won't mind any extra work involved in reformatting your manuscript at this point.

Reviewer 1

© 2026. Published by The Company of Biologists under the terms of the Creative Commons Attribution License (https://creativecommons.org/licenses/by/4.0/).

Comments for the author

The authors propose that Ewing sarcoma cells carrying EWSR1-FLI1 and reduced/lost wild-type EWSR1 are sensitive to the microtubule-destabilizing agent nocodazole. They evaluate this using three Ewing sarcoma cell lines, A673, RD-ES, and SK-ES1, as well as an inducible DLD-1 model that allows tet-on expression of EWSR1-FLI1 and auxin-mediated depletion of EWSR1 from one allele. This system provides a potentially useful approach for dissecting the mechanism underlying nocodazole sensitivity. The authors further propose that selective sensitivity to microtubule-destabilizing agents (MDAs), rather than microtubule-stabilizing agents (MSAs), is attributable to increased mitotic spindle damage.

The manuscript needs major revisions, but still seems to have a plausible conclusion. An understanding of why Ewing sarcoma is selectively sensitive to MDAs rather than MSAs is important.

1.Line 24 (1st sentence of the manuscript): "due to its activity" should be "due to their ability".

2."Vinka" should be "Vinca"

3.Line 55 "The apoptosis rate of the nocodazole-treated Ewing sarcoma cells (A673, RD-ES and SK-ES1) was significantly higher than that of MSC, supporting the previous observation of Ewing sarcoma sensitivity to MDAs (Fig 1A) [5]."

*This is not supported by Fig. 1A, because nocodazole is being treated as a class of drugs (MDAs) rather than a single-drug. I recommend the authors test 2-5 more drugs of this class, or alternatively, soften the conclusion language to make this nocodazole-specific.

4.Line 59 "Importantly, nocodazole treatment in MSC did not induce a significant increase in apoptosis, suggesting a nocodazole sensitivity specific to Ewing sarcoma (Fig 1A)."

*To support the claim this is not significant, you need a comparison bar of untreated MSC vs. nocodazole-treated MSC, showing "N.S.", if that is indeed what the statistical test shows.

*The data do not support this being "specific to Ewing sarcoma" as written because that would suggest other non-Ewing sarcoma models were tested. The language should reflect this and not overclaim.

5.The title should be revised for precision. "Loss of one EWSR1 allele" is somewhat ambiguous, because the relevant biological state is reduced wild-type EWSR1 level in the context of EWSR1-FLI1 expression. I recommend revising the title to something like "EWSR1-FLI1 expression combined with monoallelic EWSR1 depletion is associated with mitotic spindle damage and nocodazole sensitivity." This would also avoid overgeneralizing beyond nocodazole.

6.Figure 3 appears to contain a labeling problem. The figure legend states that MSC, A673, RD-ES, and SK-ES1 cells were analyzed, but the x-axis labels in the quantification panel appear to correspond to the inducible DLD-1 conditions: DMSO, EWSR1 KD, EWSR1-FLI1, and EWSR1 KD + EWSR1-FLI1. This needs to be corrected, because the current figure makes it difficult to evaluate the conclusion that Ewing sarcoma cell lines have higher levels of GTP-tubulin than MSC.

7.The manuscript treats increased GTP-tubulin signal as direct evidence of mitotic spindle damage. This interpretation may be reasonable, but it should be framed more carefully. GTP-tubulin signal can reflect altered microtubule dynamics, repair, rescue events, or lattice state. The authors should either provide orthogonal validation of spindle damage or soften the language so that GTP-tubulin is described as a marker consistent with increased spindle damage rather than definitive proof of damage.

8.The image quantification methods are underdescribed. The authors should describe how spindles were selected in greater detail.

9.The inducible DLD-1 model should be validated more clearly within this manuscript. The authors should show the degree of EWSR1 depletion and EWSR1-FLI1 induction under the AUX/DOX

© 2026. Published by The Company of Biologists under the terms of the Creative Commons Attribution License (https://creativecommons.org/licenses/by/4.0/).

conditions used for the apoptosis and microscopy experiments, ideally by immunoblotting or quantitative fluorescence.

Overall, the conclusions from this manuscript as-stated are not well-supported by the data, but the gist is plausible. This manuscript will be strengthened with a combination of edits to the language (e.g., not referencing nocodazole as a representative of all MDAs) and/or further experiments (e.g., testing more MDAs and cell lines to help support their claims).

Reviewer 2

Comments for the author

The manuscript by Schulz and Azuma addresses a significantly relevant clinical observation in pediatric oncology: the unique vulnerability of Ewing sarcoma cells to microtubule-destabilizing agents (MDAs), such as vincristine, alongside their relative insensitivity to microtubule-stabilizing agents (MSAs). The conceptual premise of the paper is compelling and addresses a long-standing mechanistic gap. However, several critical limitations regarding model lineage translatability, low imaging sample sizes, and potential statistical pseudoreplication must be resolved before the manuscript can be accepted for publication.

Major Revisions & Methodological Concerns

A. Cell Line Lineage and Background Limitations

The vast majority of the mechanistic synergy data relies strictly on the dual-engineered DLD-1 cell system. DLD-1 is a near-diploid colorectal adenocarcinoma epithelial line. While it represents an elegant tool for CRISPR-based tagging and rapid conditional manipulation, epithelial cancer cell lines possess fundamentally different baseline metabolic networks, lineage constraints, and mitotic checkpoint sensitivities than mesenchymal stem cells (MSCs) or osteo-chondrogenic lineages where Ewing sarcoma arises.

Recommendation: The authors must explicitly expand their Discussion section to address this limitation. They need to justify why an epithelial colorectal carcinoma background serves as a reliable surrogate for a pediatric bone tumor cell-of-origin context, and address how tissue-specific factors might temper or influence these findings.

B. Critically Low Microscope Sample Size

In the figure legend for Figure 3, the authors state: "The intensity of the signals from a minimum of five cells was quantified from n=3 experiments." This equates to a cohort potentially as small as 15 total cells tracked per patient-derived cell line across the entire dataset. In immunofluorescence intensity analysis, especially when drawing definitive, population-wide conclusions about metaphase spindle architecture across highly heterogeneous cells, a sample size this small is vulnerable to selection bias and spatial outliers.

Recommendation: The authors must scale up their imaging datasets. A minimum of 20-30 metaphase spindles should be captured and analyzed per experimental replicate across the three independent runs to establish statistical robustness.

C. Data Transparency & Potential Pseudoreplication

The statistical descriptions provided for the imaging data in Figures 2 and 3 lack clarity regarding the exact unit of analysis fed into the multi-comparison tests (ANOVA/Tukey's HSD). It is unclear whether the software treated each individual cell measurement as an independent value ($N \geq 15$ or $N \geq 60$) or if the technical repetitions were nested and compressed down to the true biological average of each independent experimental container (n=3).

Recommendation: To ensure the analysis does not suffer from technical pseudoreplication (which artificially deflates standard error and engineers hyper-significant p-values), the authors must

© 2026. Published by The Company of Biologists under the terms of the Creative Commons Attribution License (https://creativecommons.org/licenses/by/4.0/).

explicitly state the exact sample units, structural nesting, and degrees of freedom utilized for all imaging statistical evaluations. Additionally, presenting individual cell data spreads as scatter dot plots rather than summarized bars would greatly enhance raw data transparency.

D. Omission of Core Methodological Details

The Materials and Methods section leaves out critical operational parameters that are vital for ensuring study reproducibility:

The exact concentrations and incubation timelines for Doxycycline (induction) and Auxin/IAA (degradation) relative to the initiation of nocodazole treatment are not completely specified.

The explicit localized region-of-interest (ROI) segmentation steps, background subtraction values, and intensity thresholding boundaries used in QuPath to define a GTP-tubulin "island" must be clearly defined, as intensity-based pixel quantification is highly operator-dependent.

E. Overly Linear Narrative and Missing Literature Context (Scholarship)

The manuscript presents a compelling, highly focused model linking EWSR1-FLI1 expression and wild-type EWSR1 loss to microtubule instability. However, the discussion is overly linear and misses an opportunity for strong scientific scholarship by failing to properly integrate or cite literature that presents alternative, complicating, or conflicting views on Ewing sarcoma drug sensitivity.

*Recommendation: The authors must expand the Discussion section to contextualize their findings within broader, fusion-independent drug sensitivity landscapes. Specifically, they should discuss published data regarding how cell-autonomous traits (such as baseline replicative stress or alternative spliceosome modifications) or in vivo bone microenvironment mechanics independently influence antimitotic drug dynamics and resistance patterns.

Minor Revisions and Technical Corrections

Check spellings and grammar. There are errors in multiple places. (e.g. change "puromycine" to "puromycin", "Alexa Flour" to "Alexa Fluor" etc.)

Reviewer's Responses to Questions

Experimental quality
Are experiments performed using appropriate methods that will answer the question (or test the hypothesis or support the observations) posed by the authors? Is the right tool used for the job?
If 'No', please indicate reasons in Comments for Author box below.
Reviewer #1:
- No

Reviewer #2:
- No

Were the data analyzed using appropriate statistical tests?
If 'No', please indicate reasons in Comments for Author box below.
Reviewer #1:
- Yes

Reviewer #2:
- No

Reproducibility
Were experiments performed using adequate number of biological replicates?
If 'No', please indicate reasons in Comments for Author box below.
Reviewer #1:
- Yes

Reviewer #2:

© 2026. Published by The Company of Biologists under the terms of the Creative Commons Attribution License (https://creativecommons.org/licenses/by/4.0/).

- No

Is there sufficient raw data to assess the rigor of the analysis?
If 'No', please indicate reasons in Comments for Author box below.
Reviewer #1:
- No

Reviewer #2:
- No

Does the methods section provide sufficient detail to permit reproducibility?
If 'No', please indicate reasons in Comments for Author box below.
Reviewer #1:
- No

Reviewer #2:
- No

Completeness
Are the manuscript's conclusions supported by the data?
If 'No', please indicate reasons in Comments for Author box below.
Reviewer #1:
- No

Reviewer #2:
- No

Scholarship
Do the authors cite and discuss the merits of data that would argue for and against their conclusion?
If 'No', please indicate reasons in Comments for Author box below.
Reviewer #1:
- Yes

Reviewer #2:
- No

Does the manuscript title & abstract accurately reflect the contents of the manuscript, without hyperbole?
If 'No', please indicate reasons in Comments for Author box below.
Reviewer #1:
- No

Reviewer #2:
- No

First revision

Author response to reviewers' comments

RESPONSE TO REVIEWERS

MS ID#: bio.062763

We would like to express our gratitude to both reviewers for providing insightful comments. The comments were logically sound, and we have incorporated their suggestions into the manuscript. Their valuable feedback has improved the quality of the manuscript. The point-by point responses are listed below.

Reviewer: 1

© 2026. Published by The Company of Biologists under the terms of the Creative Commons Attribution License (https://creativecommons.org/licenses/by/4.0/).

1. Line 24: "due to its activity" was changed to "due to their ability".

2. Vinka" was corrected to "Vinca".

3. Line 55: Thank for your great suggestion. We agree that our original description was misleading. We have revised the sentence to "The apoptosis rate of the nocodazole- treated Ewing sarcoma cells (A673, RD-ES and SK-ES1) was significantly higher than that of MSCs, suggesting that the Ewing sarcoma cells are more sensitive to nocodazole (**Fig 1A**)".

4. Line 59 & Fig 1A: We agree that the phrase "specific to Ewing sarcoma" was misleading. Therefore, we revised the sentence to "highlighting the difference in nocodazole sensitivity between Ewing sarcoma and MSCs". We also added a comparison bar between untreated MSC vs. nocodazole-treated MSC, showing that the difference was not significant (N.S.).

5. Thank you for the suggestion. We changed the title to "EWSR1-FLI1 expression combined with monoallelic EWSR1 depletion is associated with mitotic spindle damage and nocodazole sensitivity". We are grateful to the reviewer, as this new title more accurately reflects the content of the manuscript than the previous version.

6. We corrected the labeling error of Fig 3. Thank you.

7. Thank you for the valuable suggestion. We agree that the usage of the GTP-tubulin as a spindle damage site could be misleading, and changed the description.

8. The image quantification procedure has now been described in the Material and Method section.

9. The description of the (*AID-EWSR1/wt: EWSR1-FLI1-mCherry/wt*) DLD-1 cell line was deposited to BioRxiv prior to the submission of this manuscript. To avoid redundant publication, we cited the preprint and included FigS1 to verify the effect of AUX and DOX.

Reviewer: 2

A. Cell Line Lineage and Background Limitations: We thank reviewer 2 for the valuable advice. We agree that the DLD-1 model has certain limitations. We have discussed the advantages and disadvantages of the model in the Discussion section.

B. Critically Low Microscope Sample Size: Thank you for the suggestion. We have now added additional sample numbers shown in Fig 3.

C. Data Transparency & Potential Pseudoreplication: We apologize for the confusing description. The exact sample units, structural nesting, and degrees of freedom used for statistical analysis have now been included in the manuscript.

D. Omission of Core Methodological Details: The timeline of AUX/DOX and Nocodazole treatment has been added to the materials and method section. In addition, the quantification method for the GTP-tubulin signals has been described in the materials and method section.

E. Overly Linear Narrative and Missing Literature Context (Scholarship): Thank you for the valuable suggestion to improve the quality of manuscript. We have included a discussion of the factors contributing to the drug resistance.

Second decision letter

MS ID#: bio.062763R1

© 2026. Published by The Company of Biologists under the terms of the Creative Commons Attribution License (https://creativecommons.org/licenses/by/4.0/).

MS Title: EWSR1-FLI1 expression combined with monoallelic EWSR1 depletion is associated with mitotic spindle damage and nocodazole sensitivity

Authors: Evan T. Schulz; Alejandro Parrales; Tomoo Iwakuma; Mizuki Azuma

Dear Dr Azuma,

I have now reached a decision on the above manuscript.

The reviewer reports are shown at the bottom of this email.

As you will see, the reviewers raised a number of substantial criticisms that prevent me from accepting the paper at this stage.

They suggest, however, that a revised version might prove acceptable, if you can address their concerns. If you think that you can deal satisfactorily with the criticisms on revision, I would be pleased to see a revised manuscript. We would then return it to the reviewers.

At this stage, we also ask you to ensure your manuscript complies with our [formatting guidelines]. Provided you are able to fully address the referees' comments, we are positive about publication of your paper (we accept over 95% of revision submissions) and therefore hope you won't mind any extra work involved in reformatting your manuscript at this point.

Reviewer 1: The authors have appropriately addressed concerns. Thank you for the detailed tracking of changes in your revised manuscript.

Reviewer 2: The authors have effectively strengthened the manuscript by incorporating detailed treatment timelines and FIJI image-processing workflows into the Methods, accurately defining statistical nesting with two-way nested ANOVAs to prevent pseudoreplication, and significantly broadening the Discussion to contextualize tubulin polymorphisms, replication stress, death pathways, and microenvironmental influences. Additionally, doubling the confocal microscopy scoring to 20-30 cells per replicate (60-90 cells total per condition) improves data rigor. However, this sample size remains on the lower boundary for quantitative microscopy, yielding modest statistical power given the high cell-to-cell variability noted between lines like A673 and RD-ES/SK-ES1. Addressing this requires either additional data collection or substantial restructuring of how those comparative findings are stated.

A key limitation remains regarding the study's scope: while acknowledging cell line limitations in the Discussion is a helpful addition, the manuscript's central claims (from the Title and Abstract to the text) still overextend by framing results from an engineered colorectal adenocarcinoma line (DLD-1) as a definitive mechanism of native Ewing sarcoma biology. To address this biological disconnect, the authors should explicitly temper their primary conclusions throughout the text to reflect that this cooperative interaction was demonstrated within a synthetic in vitro model system.

Reviewer's Responses to Questions

Experimental quality
Are experiments performed using appropriate methods that will answer the question (or test the hypothesis or support the observations) posed by the authors? Is the right tool used for the job?
If 'No', please indicate reasons in Comments for Author box below.
Reviewer #1:

- Yes

Reviewer #2:

© 2026. Published by The Company of Biologists under the terms of the Creative Commons Attribution License (https://creativecommons.org/licenses/by/4.0/).

- No

Were the data analyzed using appropriate statistical tests?
If 'No', please indicate reasons in Comments for Author box below.
Reviewer #1:
- Yes

Reviewer #2:
- Yes

Reproducibility
Were experiments performed using adequate number of biological replicates?
If 'No', please indicate reasons in Comments for Author box below.
Reviewer #1:
- Yes

Reviewer #2:
- Yes

Is there sufficient raw data to assess the rigor of the analysis?
If 'No', please indicate reasons in Comments for Author box below.
Reviewer #1:
- Yes

Reviewer #2:
- Yes

Does the methods section provide sufficient detail to permit reproducibility?
If 'No', please indicate reasons in Comments for Author box below.
Reviewer #1:
- Yes

Reviewer #2:
- Yes

Completeness
Are the manuscript's conclusions supported by the data?
If 'No', please indicate reasons in Comments for Author box below.
Reviewer #1:
- Yes

Reviewer #2:
- Yes

Scholarship
Do the authors cite and discuss the merits of data that would argue for and against their conclusion?
If 'No', please indicate reasons in Comments for Author box below.
Reviewer #1:
- Yes

Reviewer #2:
- Yes

© 2026. Published by The Company of Biologists under the terms of the Creative Commons Attribution License (https://creativecommons.org/licenses/by/4.0/).

Does the manuscript title & abstract accurately reflect the contents of the manuscript, without hyperbole?
If 'No', please indicate reasons in Comments for Author box below.
Reviewer #1:

- Yes

Reviewer #2:

- No

Second Revision

Author response to reviewers' comments

RESPONSE TO REVIEWERS

MS ID#: bio.062763

We would like to express our gratitude to both reviewers for providing thorough reviews. We believe that our latest manuscript improved its quality by incorporating their suggestions.

Reviewer: 1

We would like to thank Reviewer 1 for providing insightful comments and support.

Reviewer: 2

Thank you for providing valuable comments. Following is the point-by-point response to the Reviewer 2.

A. Critically Low Microscope Sample Size: We have now added additional sample numbers shown in Fig 2. Thank you for providing a valuable comment.

B. Limitation of the (*AID-EWSR1/wt: EWSR1-FLI1-mCherry/wt*) cell line: We would like to express our gratitude to Reviewer 2 for providing a valuable comment. We agree with the concern raised by Reviewer 2 regarding the difference in cell lineage between DLD-1 and Ewing sarcoma. In the revised text, the data obtained from the (*AID-EWSR1/wt: EWSR1-FLI1-mCherry/wt*) DLD-1 are presented as a potential model that may reflect the activities resulting from monoallelic loss of EWSR1 and expression of EWSR1-FLI1, rather than a definite conclusion.

Third Decision Letter

MS ID#: bio.062763R2

MS Title: EWSR1-FLI1 expression combined with monoallelic EWSR1 depletion is associated with mitotic spindle damage and nocodazole sensitivity

Authors: Evan T. Schulz; Alejandro Parrales; Tomoo Iwakuma; Mizuki Azuma

Dear Dr Azuma,

© 2026. Published by The Company of Biologists under the terms of the Creative Commons Attribution License (https://creativecommons.org/licenses/by/4.0/).

I am happy to tell you that your manuscript has been accepted for publication in Biology Open, pending our standard publication integrity checks. It was accepted on 10th September 2026.

© 2026. Published by The Company of Biologists under the terms of the Creative Commons Attribution License (https://creativecommons.org/licenses/by/4.0/).
